# Presentation of Ocular Syphilis in a HIV-Positive Patient with False-Negative Serologic Screening

**DOI:** 10.1155/2019/8191724

**Published:** 2019-02-03

**Authors:** Mahsaw N. Motlagh, Cameron G. Javid

**Affiliations:** ^1^University of Arizona College of Medicine, Tucson, AZ, USA; ^2^Department of Ophthalmology, University of Arizona College of Medicine, Tucson, AZ, USA

## Abstract

**Purpose:**

The ocular sequelae of syphilis are devastating and may cause blindness. The ambiguous nature of its ocular manifestations makes syphilis difficult to detect. Though uncommon, the rise of syphilis in the United States requires a renewed understanding of its ophthalmic presentation to prevent devastating outcomes. We present this case to raise awareness for the increasing prevalence of ocular syphilis and appropriate serologic testing.

**Observations:**

We describe a 65-year-old HIV-positive male with worsening retinitis, uveitis, and rapid visual loss. Initial lab results showed a nonreactive rapid plasma reagin (RPR) for syphilis. However, subsequent *Treponema pallidum* antibody testing was positive 48 hours after initial false-negative serologic screening. The patient had a rapid and successful recovery following treatment with penicillin.

**Conclusions and Importance:**

The incidence of syphilis is on the rise once again, and patients living with HIV are at increased risk. Ocular syphilis should be considered in susceptible populations in the clinical setting of retinitis, uveitis, and worsening visual loss with unknown cause. In addition, retesting for syphilis will decrease the prevalence of false-negative results, especially in patients with high clinical suspicion.

## 1. Introduction

Syphilis has a notorious reputation as the great masquerader. Caused by the spirochete bacterium *Treponema pallidum*, syphilis has a myriad of typical and atypical presentations. With syphilis rates on the rise in the United States, it is important to be aware of ocular symptoms [1]. In this case, described below, a 65-year-old HIV-positive male presents with worsening visual loss and a negative RPR. Furthermore, the repeated testing for treponemal antibodies was positive, promoting the importance of repeat testing in patients with high clinical suspicion.

## 2. Case Report

**Figure 1:**
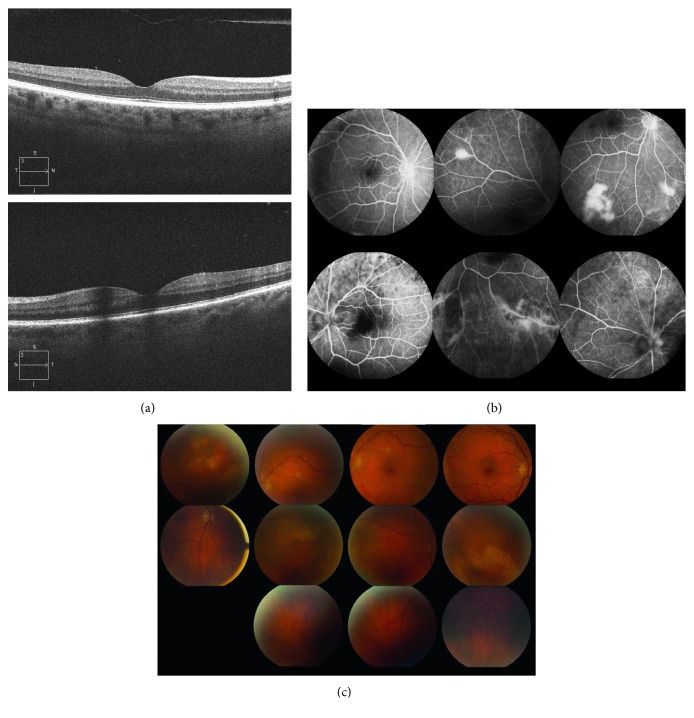
Baseline imaging results. (a) Optical coherence tomography upon initial presentation was unremarkable. (b) Fluorescein angiography at the first visit revealed focal hyperfluorescence inferior to the optic nerve in the right eye (*top*) and vasculitis of the inferotemporal vessels in the left eye (*bottom*). (c) Fundus photos upon initial presentation. Normal retinal vessels in the right eye. Peripheral retinitis with white yellow necrotic retina and slight disc edema in the left eye.

A 65-year-old male with a past medical history positive for HIV was referred for ophthalmic consultation for new onset flashes and floaters in the left eye. The patient also reported decreased and blurry vision in the left eye. At the initial time of presentation, the visual acuity test revealed 20/20 in the right eye and 20/50 in the left eye. Optical coherence tomography (OCT) findings were unremarkable (Figure 1(a)). Fluorescein angiography (FA) revealed focal hyperfluorescence in the right eye and vasculitis in the left eye (Figure 1(b)). Fundus photography was unremarkable for the right eye but revealed peripheral retinitis, slight disc edema, and 2+ vitritis in the left eye (Figure 1(c)). The patient's CD4 count was >600 cells/mm^3^, and viral load was undetectable. At this point, the clinical presentation suggested acute retinal necrosis (ARN), and a sample of anterior fluid was sent to be analyzed for varicella-zoster virus (VZV), herpes simplex virus (HSV), and cytomegalovirus (CMV). The patient was started on prophylactic valacyclovir and requested for follow-up in 2 days.

Forty-eight hours later, the patient's visual acuity had decreased to 20/60 in the left eye, but no changes were noted in the right eye. The remainder of the physical exam was unremarkable, and the patient was again scheduled for follow-up one week later.

The following week, just 8 days after initial presentation, the patient presented with dramatically worsened vision. His acuity was still preserved in the right eye at 20/25 but had decreased to 20/200 in the left eye. The concern grew stronger as the PCR results returned negative for HSV-1, HSV-2, VZV, and CMV. Despite worsened visual acuity on exam, the patient's vitritis and retinitis improved, further complicating the case. Given this clinical picture, the patient was started on 30 mg prednisone daily in addition to the previously prescribed valacyclovir. The patient was then requested for follow-up appointment in 2 weeks.

**Figure 2:**
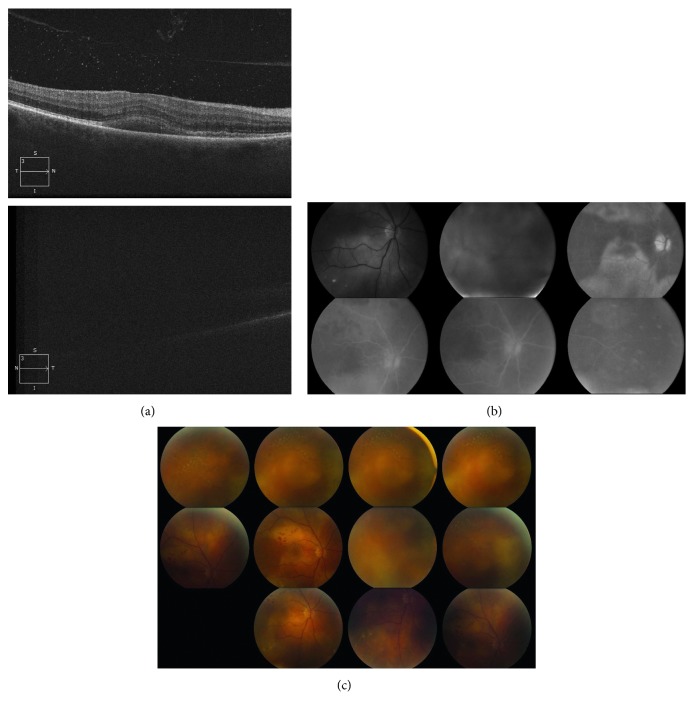
Imaging results 21 days after initial presentation. (a) Optical coherence tomography of the right eye (*top*) showed vitreous debris with no evidence of subretinal fluid. The view of the left eye (*bottom*) was limited due to heavy vitreous debris. (b) Fluorescein angiography following visual decline. There are marked retinitis and vitreous debris in the right eye (*top*) and limited views due to heavy vitreous debris in the left eye (*bottom*). (c) Fundus photos revealed vitreous debris with 1+ vitritis and peripheral retinitis in the right eye. Heavy vitreous debris with 4+ vitritis causing limited views in the left eye.

**Figure 3:**
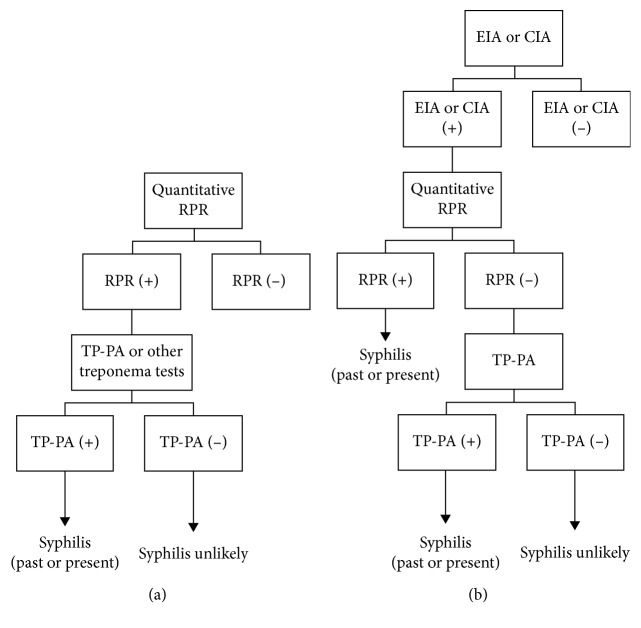
Syphilis screening algorithms. Traditional serologic screening (a) compared to reverse sequence serologic screening (b). Screening algorithms adopted from CDC recommendations [3, 4]. RPR, rapid plasma reagin; TP-PA, *Treponema pallidum* particle agglutination; EIA, enzyme immunoassay; CIA, chemiluminescence immunoassay.

At the next follow-up visit, 20 days after initial presentation, it was apparent that the patient's ophthalmologic condition was progressively worsening. Visual acuity in the right eye was now decreased to 20/150. In the left eye, the patient could only recognize hand motion. Expectedly, OCT, FA, and fundus views were all limited in the left eye due to worsening of the vitreous debris (Figure 2). From limited views, physical exam revealed 1+ vitritis in the right eye and 4+ vitritis in the left eye (Figure 2(c)). FA of the right eye showed retinitis superiorly and inferiorly, which extended into the macula (Figure 2(b)). Both eyes were affected by posterior uveitis. Despite the worsening condition of the patient, serum testing obtained by the referring infectious disease specialist was all unremarkable. This included a nonreactive RPR screen with reflex titer for syphilis. At this point, it was discussed with the patient that visual prognosis was considered extremely poor. Even with the negative screening results from the referring physician, there was still suspicion for syphilis as the patient was at considerable risk. It is well known that a nonreactive RPR test does not rule out syphilis infection; therefore, it was decided to repeat serologic testing. However, this time, the patient was tested using the alternative serologic screening algorithm for syphilis, which tests directly for treponemal antibodies (Figure 3) [2, 3].

Follow-up testing with the alternative method was reactive. Additionally, the RPR titer was now high, confirming the diagnosis of syphilis (Table 1). The patient was admitted on the same day for treatment with IV penicillin. The patient's vision dramatically improved, and he was discharged the following day on a 14-day course of penicillin. At one-week after treatment, the patient's acuity had progressed to 20/40 with pinhole improvement to 20/30 in the right eye and 20/50 with pinhole improvement to 20/30 in the left eye. A summary of the patient's visual acuity decline and subsequent improvement following treatment are found in Table 2. The repeat RPR testing promoted the significance of understanding the available serologic tests for syphilis. Had an antibody test been ordered initially, it likely would have yielded positive results. In addition, if repeat testing was not conducted for RPR, the diagnosis for this patient with extremely poor prognosis would have been delayed, potentially resulting in blindness. The aggressive nature of ocular syphilis is unforgiving, but with a quick and accurate diagnostic tool, simple treatment with IV penicillin yields excellent outcomes (Table 2).

## 3. Discussion

Syphilitic uveitis remains a rare condition in the United States [5]. However, in conjunction with the continued increase in prevalence, it is imperative to remain aware of the ophthalmic diseases associated with syphilis [1, 6]. Uveitis is the most common ophthalmologic presentation of syphilis and can appear at any course of the disease [7, 8]. The diagnosis of ocular syphilis should be considered in any patient with unexplained uveitis or vision loss, especially in patients with comorbid risk factors such as HIV and men who have sex with men [9]. The prognosis of this rare complication of syphilis, if left untreated, is extremely poor and can lead to complete vision loss [10, 11]. Thus, in consideration of the above, we recommend setting a low threshold for considering syphilitic uveitis in high-risk populations.

More notably, this case in particular promotes the importance of considering a false-negative screening result. For a patient with high clinical suspicion of syphilis, retesting is warranted. Though long-held practice recommends serologic screening with a nontreponemal test, most commonly RPR or Venereal Diseases Research Laboratory (VRDL) test, followed by confirmatory treponemal testing, it may be time to consider an alternative screening algorithm as the standard of care. In fact, many laboratories have begun adopting a reverse sequence of screening, in which a treponemal enzyme and chemiluminescence immunoassay (EIA/CIA) is used first, followed by a nontreponemal test for confirmation (Figure 3) [3]. This would gradually eliminate the need for repeat testing, as the treponemal antibody testing has no false-negative results [3]. This paradigm shift requires better understanding from the clinician of the serologic screening that is available.

The RPR test does not measure the bacterium directly, but instead measures antibodies to lipoidal material released from damaged host cells [12]. Therefore, the possibility of false-negative results remains plausible during any period within the course of the disease. Moreover, a reactive nontreponemal test does not confirm *Treponema pallidum* infection [12]. This discrepancy, in the context of acute ocular syphilis, warrants the use of an alternate algorithm that seeks the most accurate and precise serologic testing first [3, 13].

## 4. Summary Statement

A 65-year-old man with HIV developed rapid visual loss with unknown cause. Serologic testing with traditional rapid plasma regain was negative. However, subsequent testing with treponemal antibody testing was positive, indicating initially false-negative results.

## Figures and Tables

**Table 1:** Serologic screening results. Upon initial presentation, the traditional testing algorithm was utilized. However, at the time of follow-up (secondary results), the alternative testing algorithm was followed.

Serologic test	Initial test results	Secondary test results (48 hours later)	Reference ranges
RPR screen	Nonreactive	Reactive	Nonreactive
RPR titer	Nonreactive	1 : 512	Nonreactive
Syphilis antibody total	N/A	6.8	≤0.8
Syphilis antibody result	N/A	Reactive	Nonreactive
*Treponema pallidum* antibody	N/A	Reactive	Nonreactive

RPR, rapid plasma reagin.

**Table 2:** Summary of visual acuity decline.

Date	OD	OS
Initial consult (day = 0)	20/20	20/50
Follow-up 1 (day = 2)	20/20	20/60
Follow-up 2 (day = 8)	20/25	20/200
Follow-up 3 (day = 20)	20/25	20/400
Follow-up 4 (day = 21)	20/150	20/400
Follow-up 5 (day = 41)	20/40 PH 20/30	20/50 PH 20/30
